# Polymeric Systems for Cancer Immunotherapy: A Review

**DOI:** 10.3389/fimmu.2022.826876

**Published:** 2022-02-22

**Authors:** Thai Minh Duy Le, A-Rum Yoon, Thavasyappan Thambi, Chae-Ok Yun

**Affiliations:** ^1^ Department of Bioengineering, College of Engineering, Hanayang University, Seoul, South Korea; ^2^ Institute of Nano Science and Technology (INST), Hanayang University, Seoul, South Korea; ^3^ Hanyang Institute of Bioscience and Biotechnology (HY-IBB), Hanyang University, Seoul, South Korea; ^4^ GeneMedicine CO., Ltd., Seoul, South Korea

**Keywords:** immune checkpoint inhibitor, immunotherapy, polymeric system, oncolytic adenovirus, CAR-T cell

## Abstract

Immunotherapy holds enormous promise to create a new outlook of cancer therapy by eliminating tumors *via* activation of the immune system. In immunotherapy, polymeric systems play a significant role in improving antitumor efficacy and safety profile. Polymeric systems possess many favorable properties, including magnificent biocompatibility and biodegradability, structural and component diversity, easy and controllable fabrication, and high loading capacity for immune-related substances. These properties allow polymeric systems to perform multiple functions in immunotherapy, such as immune stimulants, modifying and activating T cells, delivery system for immune cargos, or as an artificial antigen-presenting cell. Among diverse immunotherapies, immune checkpoint inhibitors, chimeric antigen receptor (CAR) T cell, and oncolytic virus recently have been dramatically investigated for their remarkable success in clinical trials. In this report, we review the monotherapy status of immune checkpoint inhibitors, CAR-T cell, and oncolytic virus, and their current combination strategies with diverse polymeric systems.

## 1 Introduction

According to the World Health Organization (WHO), cancer is among the top leading causes of nearly 10.0 million deaths worldwide in 2020 (1, 2). Even though traditional treatment methods, such as invasive surgery, chemotherapy, targeted therapy, and radiation, have prevailed and made tremendous progress in the clinical setting, these regimens still face some inherent limitations in terms of therapeutic efficacy and safety (3–6). To address these issues, cancer immunotherapy, also known as immuno-oncology, has stepped into the spotlight and many kinds of immune therapeutics are investigated in the research and development stage (7, 8). Some of them have even been commercialized (9, 10). Cancer immunotherapy is a type of biological therapy that utilizes the body’s immune system to generate the attacking response of the tumor cells and thus produce an anti-tumor effect (11, 12). It can train the immune system to recognize and strike specific cancer cells and boost immune cells to help them eliminate cancer. It is noteworthy that cancer immunotherapy targets not only the primary tumor but also the secondary tumor metastasis by stimulating systemic immune response (13, 14). Further, it can inhibit tumor recurrence through cancer-specific memory immune response which will be reactive when encountering tumor associate antigens (TAA) (15, 16).

There have been diverse cancer immune therapeutics, such as cancer vaccines, antibody therapy, cytokines, immune checkpoint inhibitors (ICI), adoptive cell transfer (ACT), and oncolytic viruses (OV) (17, 18). Among those immune therapeutics, remarkable success in the commercialization of ICI and chimeric antigen receptor (CAR)-T cells have driven cancer immunotherapy into the limelight (19–22). ICI, which blocks the binding of checkpoint proteins with their partner proteins and allows T cells to kill cancer cells, has been revolutionarily developed, since Yervoy, the first ICI and CTLA-4 inhibitor, was approved by the U.S. FDA in 2011. CAR-T is a genetically engineered T cell to express artificial T cell receptors and specifically target tumor cells. In addition to ICI and CAR-T, the utilizations of OV have rapidly expanded in the past few years since the U.S. FDA approved the first OV, Imlygic, in 2015 (23). OV has been recognized as a novel therapeutic platform due to its unique feature, selectively replicating in and eradicating cancer cells through a domino-like cascading infection of tumor cells, which other conventional therapeutics cannot mimic (24). Moreover, it is also highlighted with its capacity to target cancer immunity in multiple steps, leading to potent clinical benefits (25, 26).

Even though these immunotherapies possess dramatic breakthroughs in the last decade, manifold obstacles regarding therapeutic efficacy and safety remain to be overcome. To further elaborate, previous research has shown that less than 50% of the solid tumor types have positive responses to ICI therapy (27). Moreover, regarding safety issues, many researchers have demonstrated the association of ICI therapy with several side effects such as colitis, fatigue, pneumonitis, endocrinopathies, and dermatitis (28, 29). Meanwhile, for the CAR-T cell therapy, safety is also still an utmost concerning issue according to neurotoxicity and severe side effects, which include death, cytokine release syndrome, hyperuricemia, hyperkalemia, acute anaphylaxis, and B-cell aplasia (30, 31). These obstacles are also found in systemically administered OV, which demonstrates limited therapeutic efficacy due to its hepatotoxicity and immunogenicity that respectively trigger nonspecific liver toxicity and inflammatory responses, making it difficult to cure inaccessible tumors (32, 33). Further, the complex tumor microenvironment (TME) is also a difficult hurdle that limits the application of current immunotherapies (34, 35). In addition, the above immunotherapies required a large dose of therapeutic drugs regarding their instability and short half-life, which exhibits toxicity and severe side effects in a certain number of patients.

**Figure 1:**
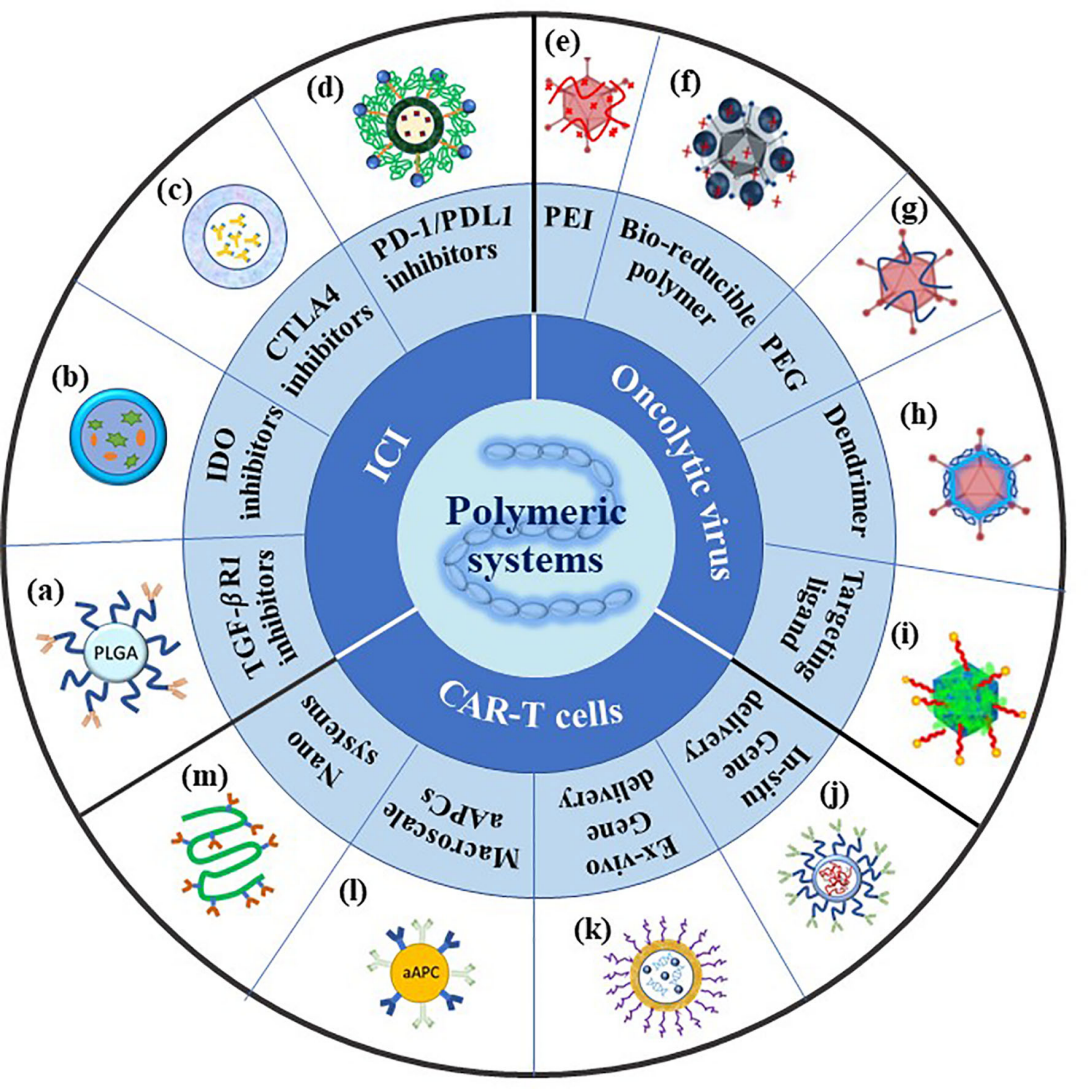
Summary of polymeric systems for cancer immunotherapy. ICI: **(A)** Scheme of antibody conjugated PEG-PLGA polymeric nanoparticles recreated referencing from (50); **(B)** scheme of NLG919(IODI)/IR780 coloaded micelles recreated referencing from (51); **(C)** scheme of polymeric micelles containing ICI antibodies recreated referencing from (52); **(D)** scheme of PEG sheddable, anti-PD-1 antibody (aPD-1)-conjugated, and PTX-loaded micelle recreated referencing from (53); OV: **(E)** Scheme of cationic PEI-Ad complex created referencing from (54); **(F)** scheme of PPSA-Ad complex reused referencing from our group (55); **(G)** scheme of PEG conjugated-Ad created referencing from (56); **(H)** scheme of amphiphilic dendrimer binding Ad recreated referencing from (57); **(I)** scheme of Ad/chitosan-PEG-FA nanocomplex reused referencing from our group (58); CAR-T cells: **(J)** Scheme of targeted mRNA-carrying polymeric nanoparticle recreated referencing from (59); **(K)** scheme of targeted pDNA-carrying supramolecular self-assemble nanoparticles recreated referencing from (60); **(L)** scheme of artificial antigen presenting cell recreated referencing from (61); **(M)** scheme of semi-stiff synthetic dendritic cells recreated referencing from (62).

To lower the negative effects and enhance the targeting of immunotherapies, advanced delivery systems have been exploited. In recent years, diverse delivery systems have been developed for immunotherapeutic drugs, both for local, systemic delivery and sustained release *in vivo* (36–38). Among these materials, diverse polymeric systems have been exploited both as excellent carriers for therapeutic immunogenic agents and favorable adjuvants. These polymeric systems obtain various advantages including superior biocompatibility and biodegradability, effective activity for immune stimulation, tunable size, designable structure, and large loading capacity for immune-inducing factors (39–43). Moreover, advanced polymeric systems with different functions can be utilized to carry immune pharmaceuticals to targeting organs by different routes of administration such as subcutaneously, intranasally, and intravenously (44–47). To elaborate, these pharmaceutical drugs are delivered through polymeric systems in various forms such as polymer–drug conjugates or drug-loaded micelles (48, 49). These efficient delivery systems are therapeutically promising due to several reasons. Most polymer backbones consist of diverse functional groups, enabling the easy conjugation of pharmaceutical drugs to polymer systems through specific ligands. Moreover, the fabricable nano size system can allow the polymeric system to stay in the blood circulation and induce passive tumor targeting. Due to these advantages, the polymeric systems are considered a promising strategy to efficiently apply for diverse immunotherapies (**Figure 1**).

In this review, we focus on representing immunotherapeutic strategies including immune checkpoint inhibitors, CAR-T cells, and oncolytic viruses, and their combined application with polymeric systems. First, we would like to introduce the recent development of immune checkpoint inhibitor strategies and then discuss relevant polymeric systems applied with each ICI type. Second, we describe adoptive cell transfer immunotherapy and its representative which is a CAR-T cell. We analyze the current manufacturing methods of the CAR-T cell and its limitations. We then demonstrate how application of polymeric systems can reduce these limitations and innovate CAR-T cell to a new clinical efficacy. Finally, we will present the ongoing possibility of the oncolytic viruses in immunotherapy. We intensify on adenoviruses which are the most extensively studied virus type. We begin by describing basic oncolytic adenovirus biology and analyze their advantages as well as their limitations. We then introduce some typical modifications with polymeric systems that can be used to promote better anti-tumor inhibition.

## 2 Different Immunotherapies and Polymeric Systems Applying for These Strategies

### 2.1 Immunological Checkpoint Inhibitors

#### 2.1.1 Monotherapy of ICI

Immunological checkpoint inhibitors currently have been dramatically investigated and the most reported inhibitors are cytotoxic T-lymphocyte-associated protein 4 (CTLA-4) and programmed death receptors 1/programmed death receptor-ligand 1 blockade (PD-1/PD-L1). The body’s immune system uses the immune checkpoints to control the corporeal immune balance and maintain self-tolerance (63). In normal conditions, the activated T cells express PD-1 to recognize abnormal or cancerous cells and then eliminate them to protect the body from their development (64, 65). However, the tumor cells might up-regulate the expression of PD-L1 or PD-L2 that bind to PD-1 to evade recognition and attack of immune cells (66, 67). Therefore, anticancer immunotherapy can be achieved by using blocking inhibitors of PD-1 or its ligand. Another immune checkpoint is CTLA-4, which diminishes the T-cell activity and assists the maintenance of self-tolerance (68). The anti-CTLA-4 antibody has been used to block the CTLA-4 to induce T cell activation, which inhibits tumor growth.

Nonetheless, many challenges remain with these checkpoint blockade strategies that limit their application for complete cancer treatment. As mentioned above, the administration of immunological checkpoint inhibitors can cause severe harm to different normal organs (69, 70). Moreover, only a small portion of patients show positive effectiveness of checkpoint inhibitor treatments, this phenomenon is still under study (71, 72). In addition, the extracellular matrix and microenvironment of different tumors suppress the immune recognition and activation (73). With the new development of polymers and biomaterials science, these challenges can be overcome by utilizing various polymers to obtain targeting tumor delivery (**Table 1**).

**Table 1:** Summary of recent research on different polymeric systems for immunological checkpoint inhibitors.

Type of immunotherapy	Polymer systems	System properties^a^	Payloads	Affected immune cell	Cancer cell model	Key findings	Ref.
Immunological checkpoint inhibitors (anti-PD1)	Folic acid (FA)–and PEG functionalized polyethylenimine (PEI) polymers	D: 167 nmZ: 22.8 mV	PD-L1 siRNA	T-cells are engineered to co-express a CAR (T1E28z) that targets the extended ErbB family	SKOV-3-Luc cells (epithelial ovarian cancer cells)	Successfully delivered PD-L1 siRNA into EOC cells and blocked PD-1/PD-L1 interactions with T cell. FA targeted EOC cell and lowered cytotoxicity of PEI	(74)
Immunological checkpoint inhibitors (TGF-βR1 inhibitor)	F(ab’)2-Mal-PEG-PLGA	D: 267-273 nm	R848, SD-208	CD8+ T cells	B16 melanoma cells, MC38 cells	F(ab’)2-Mal-PEG-PLGA targeted specific T cell subsets and functionally neutralized co-inhibitory receptors	(50)
Immunological checkpoint inhibitors (TGF-βR1 inhibitor)	PEG5k–PLA11k and BHEM-Chol	D: 141.6 ± 6.1 nm	CTLA-4 siRNA	CD4+ T cells, CD8+ T cells, Tregs	B16 melanoma cells	Nanoparticles efficiently delivered siRNA into T cells; increased the number and percentage of effector CD4+ T cells and CD8+ T cells and decreased the ratio of CD4+ FOXP3+ Tregs	(75)
Immunological checkpoint inhibitors (IDO inhibitor)	MPEG-PCL	D: 43 ± 3.2 nm	IR780, NLG919	T cells, Tregs	MCF-7, 4T1 breast cancer cells	NLG919/IR780 micelles inhibited the activity of IDO, accumulated in the tumor site *via* passive targeting and migrated to the lymphatic system, increased the infiltrated T cells in tumor tissue	(51)
Immunological checkpoint inhibitors (IDO inhibitor)	PEG2k-Fmoc-NLG	D: ~100 nm	PTX	CD8+T cells	4T1.2 breast cancer cells, B16 melanoma cells	PEG2k-Fmoc-NLG alone enhanced T-cell immune responses. Systemic delivery of paclitaxel (PTX) using the PEG2k-Fmoc-NLG nanocarrier improved antitumor response in both breast cancer and melanoma mouse models	(76)
Immunological checkpoint inhibitors (IDO inhibitor)	POEG-b-PSSNLG prodrug (PSSN10)	D: 134.7 - 175.1 nm	DOX	CD4+ Tcells, CD8+ T cells, Tregs, G-MDSCs, M1, M2	4T1.2 breast cancer cells	PSSN10 efficiently delivered both NLG and DOX to the tumor tissue. PSSN10 stimulated higher percentage of functional T cells (CD4+ and CD8+) and lowered percentages of Treg cells and MDSCs with DOX or DOXIL	(77)
Immunological checkpoint inhibitors (IDO inhibitor)	POEG-b-PVBIND	D: 17.90 ± 0.45 nm and 50.83 ± 1.25 nm (with DOX)Z: -1.23 ± 1.25 mV and -2.34 ± 2.48 (with DOX)	DOX	CD8+ T cells	4T1.2 breast cancer cells	Dox-triggered ICD promoted intra-tumoral infiltration of CD8+ T cells and IFN-c-production by CD8+ T cells. Cleaved indoximod significantly increased CD8+ T cell infiltration while reducing the immunosuppressive T regulatory cells (Tregs)	(78)
Immunological checkpoint inhibitors (IDO inhibitor)	PEG2k-Fmoc-1-MT prodrug	D: 164.3 - 298.4 nm.Z: −0.237 to 0.672 mV	DOX	CD4+ Tcells, CD8+ T cells, Tregs	4T1 breast cancer cells	PEG2k-Fmoc-1-MT prodrug inhibited ability of IDO and effectively deliver DOX and 1-MT totumors, subsequently enhancing immune responses	(79)
Immunological checkpoint inhibitors (IDO inhibitor)	PEG-P(MLT) Block copolymer	D: ~80 nm	None	THP-1 cells	None	PEG-P(MLT) can release active MLT after enzymatic degradation, toward establishing superior antitumor immunotherapies	(80)
Immunological checkpoint inhibitors (anti-CTLA-4)	pLHMGA	D: 11-15 mm	Anti-CTLA-4, CD40 agonistic antibody	No data	MC-38 cells	pLHMGA microparticles excellently delivered CTLA-4 and CD40 and provided long-lasting and non-toxic antibody therapy for immunotherapy of cancer	(81)
Immunological checkpoint inhibitors (anti-CTLA-4)	H-2Kb/TRP2-IgDimer-antiCD28 coupled PLGA- microparticles	D: 4.5 ± 1.2 µmZ: 36.2 ± 5.6 mV	IL-2, anti-CTLA-4	CD8+ T cells	B16 melanoma cells, S180cells	PLGA-microparticles sustained co-release of IL-2 and anti-CTLA-4, synergistic effects in activating and expanding tumor antigen-specific T cells both *in vitro* and *in vivo*	(82)
Immunological checkpoint inhibitors (anti-CTLA-4)	m-dextran based nanoparticles	D: 250 nm	Anti-CTLA-4, anti-PD1	CD4+ Tcells, CD8+ T cells	B16F10 cells	The nanoparticles of a-PD1 released in a sustained manner. The co-delivery aCTLA-4 and aPD1 system resulted in synergistic treatment of melanoma	(83)
Immunological checkpoint inhibitors (anti-PD1)	1-MT-conjugated hyaluronic acid (m-HA)	D: 151 nmZ: -17.1 ± 0.2 mV	Anti-PD1	CD4+ Tcells, CD8+ T cells, Tregs	B16F10 cells	The synergistic therapy with microneedle sustained release enhances retention of checkpoint inhibitors in the tumor microenvironment	(52)
Immunological checkpoint inhibitors (anti-PD1)	Azide-PEG-PAsp(Dip/Bz)	D: 128.7 ± 10.1 nmZ: -4.7 ± 0.7 mV	PTX, anti-PD1	CD8+ T cells	B16F10 cells	The micelle could control the release of aPD-1 and PTX by responding to the MMP-2 being enriched in tumor tissue and lysosomal acidity of tumor cells	(53)

^a^D, diameter; Z, Zetapotential.

#### 2.1.2 Combination ICI Therapy With Polymer Systems

One of the typical strategies in ICI is applying siRNA gene transfection to express the protein knockout of the PD-1/PD-L1 immunosuppressive pathway. This strategy has performed promising results for cancer treatment (84). Specifically, even though adoptive T cell immunotherapy performs promising results in epithelial ovarian cancer (EOC) treatment, the EOC cell-expressed PD-L1 can interact with PD-1 from T cells and induce undesired immunosuppression resulting in low therapeutic effect (85). To overcome this problem, Teo et al. have designed a folic acid (FA) functionalized PEI polymer complexed with PD-L1 siRNA. The research showed the polyplex, which consisted of FA/polymer/siRNA, has successfully blocked the PD-1 and PD-L1 pathways and repelled the immunosuppression of T cells, leading to promote the recognition of T cells toward EOC cells (74). It is important that the FA not only lower the cytotoxicity of the PEI but also effectively enhance specificity of uptake into EOC tumor cells.

On the other hand, transforming growth factor-β (TGF-β) mediated tumor microenvironment also plays an important role in immune suppression besides the PD-1/PD-L1 pathway (86, 87). Unfortunately, the TGF-β signaling is necessary for many cellular processes so utilizing TGF-βR1 inhibitors can cause severe side effects such as hemorrhagic, degenerative, and inflammatory lesions in heart valves (88). To overcome this problem, diverse polymeric nonviral vectors have been generated to encapsulate TGF-βR1 inhibitors and target them to the tumor site. In 2017, Schmid et al. introduced a CD8+ T cell-specific nanoparticle system based on PLGA-PEG polymer conjugated with anti-CD8a F(ab’)2 fragments to encapsulate TGF-βR1 inhibitor SD-208 (50). The nanoparticles system successfully reduced the cytotoxicity of TGF-βR1 inhibitor SD-208 to normal cells and recover the immune function of T cells. Moreover, the PD-1-PLGA-PEG nanoparticle co-delivery of Toll-like receptor (TLR7/8) agonist (R848) showed recruited a significantly high number of T-lymphocytes at the tumors. In addition, a nanoparticle system containing poly(ethylene glycol)-block-poly(D,L-lactide) (PEG5k–PLA11k) and the cationic lipidN,N-bis(2-hydroxyethyl)-N-methyl-N-(2-cholesteryoxycarbonyl-aminoethyl) ammonium bromide (BHEM-Chol) has been used to deliver immunosuppressive factor siRNA to a tumor (75). The siRNA encapsulated in the copolymer-based nanoparticles not only was protected from enzymatic degradation but also enhanced the cell internalization compared with negatively charged bare siRNAs. The system exhibited a favorable modulation implement in tumor invasive CTL. The loaded CTL-associated molecule-4-siRNA nanoparticles (NPsiCTLA-4) effectively stimulate T cells’ activation and hinder tumor growth in melanoma mice.

To overcome the limitations of checkpoint blockers, the polymeric micelles have also been a promising system. According to their special structure which consists of polymeric amphiphiles, micelles can carry and deliver manifold hydrophobic drugs to the target tumor. Recently, Peng et al. introduced a polymeric micelle system for tumor immunity post photothermal therapy (PTT) based on amphipathic polymer MPEG-PCL to co-deliver photosensitizer IR780 and NLG919 (an indoleamine 2,3-dioxygenase (IDO) inhibitor) (51). This nano system has shown sufficient accumulation at the tumor site and shifts to lymph nodes to promote the activation of T lymphocytes. In another research, an immunostimulatory dual-functional nanocarrier based on a prodrug conjugate of PEG with NLG919 was studied by Chen et al. The system was also equipped with a Fmoc group, a drug-interactive motif for enhancing drug loading capacity and formulation stability. The PEG2k-Fmoc-NLG alone showed greatly stimulated T-cell immune responses and excellent tumor inhibition *in vivo*. It is noteworthy to mention that the systemic administration of paclitaxel (PTX) loaded PEG2k-Fmoc-NLG nano system exhibited significant tumor inhibition in both melanoma and breast cancer mouse models (76). A redox-responsive immunostimulatory polymeric prodrug carrier which can controllably co-deliver chemotherapeutic DOX and immune checkpoint inhibitor NLG was introduced by Sun et al. (77). The system, which is called POEG-b-PSSNLG prodrug (PSSN10), was a synthesized poly(oligo(ethylene glycol) methacrylate)-poly(N,N′-(tbutyoxycarbonyl)cystamine) copolymer conjugated NLG919 prodrug which can self-assemble into nano-sized micelles. The PSSN10 carrier can improve cell immune responses in the lymphocyte-Panc02 co-culture experiments and exhibited significant anti-tumor activity *in vivo*. Moreover, the DOX/PSSN10 micelles displayed higher efficacy in the tumor growth inhibition and longer survival rate of 4T1.2 tumor-bearing mouse model compared with free DOX or a clinical formulation of liposomal DOX (DOXIL). Another IDO inhibitor, indoximod, was also co-delivered with DOX in a synthesized copolymer POEG-b-PVBIND micelles by Wan et al. in 2019 (78). The indoximod conjugating copolymer micelles effectively promoted the anti-tumor immunity resulting in an extreme tumor inhibition effect in a preclinical breast cancer model. In a different concept, Lan et al. developed a dual functional indoximod-based carrier PEG2K-Fmoc-1-MT also for breast cancer chemo and immunochemotherapy (79). The polymeric micelle itself successfully inhibited IDO effect with decreased kynurenine (KYN) production leading to the proliferation of CD4+ and CD8+ T cells. The DOX/PEG2kFmoc-1-MT micelles can generate an immunogenic cell death process, subsequently secreting many cytokines [such as interferon (IFN)-γ, IL-2, and tumor necrosis factor-alpha (TNF-α)] inducing later T cell-mediated immunity. The 4T1 murine breast cancer model tumor inhibition profile of DOX/PEG2kFmoc-1-MT micelles was dramatically high with long survival time compared with other groups. Another amphiphilic PEGpoly-1-Methyl-l-Tryptophan (MLT) block of copolymer self-assembled polymeric micelles was investigated by Huang et al. which showed effectively reduced levels of KYN in activated macrophages (80).

As mentioned above, CTL-4 can suppress the activation of T cells and promote self-tolerance. The anti-CTLA-4 antibody can be used for blocking the CTLA-4 and stimulating the activity of T cells toward the tumor. CTLA-4 antibodies have been loaded in poly(lactic-co-hydroxymethyl-glycolic acid) (PLHMGA) by Sima et al. for cancer immunotherapy. The nanoparticle system has been proven to block inhibitory receptors on T cells and obtained promising therapeutic efficacy than the IFA formulation in colon carcinoma tumor model (MC-38) (81). Lei Zhang et al. reported a study on PLGA microparticles to co-deliver IL-2 and CTLA-4 antibodies. The surface of the PLGA microparticles was conjugated with a H-2Kb/TRP2-Ig dimer and anti-CD28. The polymeric macroparticles successfully co-released IL-2 and anti-CTLA-4 inducing dual effects in activating and promoting tumor antigen-specific T cells. The systems exhibited enhancement in anti-tumor efficacy in a mouse melanoma model (82).

**Figure 2:**
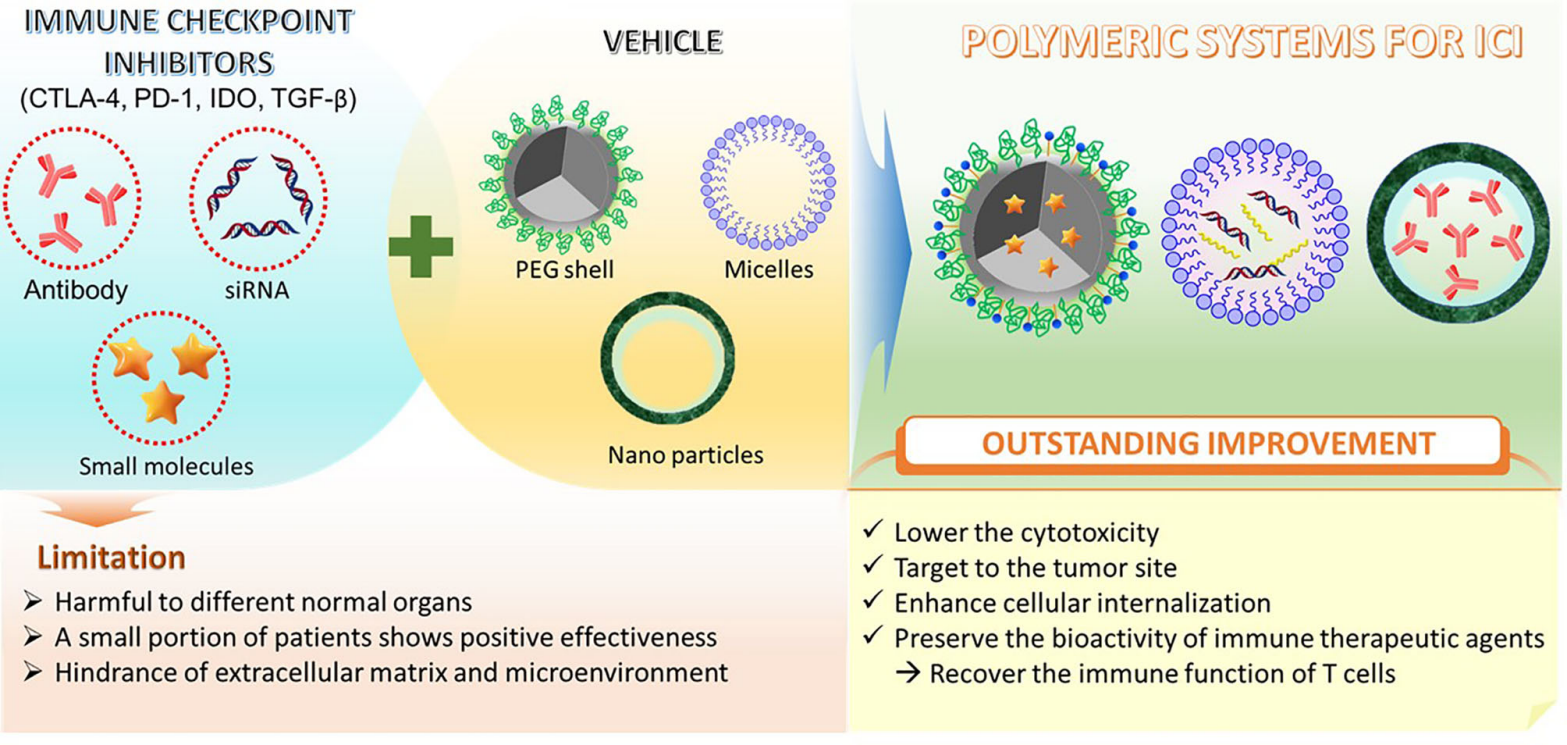
Schematic of different polymeric systems designed for ICI therapy.

Similar to CTL-4, diverse PD-1/PDL-1 inhibitors have been used for combinatorial therapy in recent clinical trials (89). Different kinds of polymers have been exploited with PD-1/PD-L1 blockade for cancer immunotherapy presently. Especially, utilizing the synergistic effect of diverse types of delivery systems can exploit the maximized potential of immune therapeutics. For example, Chao et al. reported a hyaluronic acid (a biocompatible natural polymer) microneedle integrated with pH-sensitive dextran nanoparticles (NPs) that can encapsulate and release PD-1 antibodies in a controlled manner to melanoma tissue. The report showed that this self-degradable microneedle encapsulated PD-1 antibody system generated a higher robust immune response compared to free anti-PD-1 antibody at the same dose in a B16F10 melanoma model (83). Another study conducted by Ye et al. has also produced immunotherapeutic nanoparticles from hyaluronic acid but modified with 1-methyl-DL-tryptophan (1MT) to deliver anti-PD-1 antibody. The particles combined with microneedle successfully sustain release and increased the accumulation of anti-PD-1 antibodies in the TME. The system indicated the improvement in tumor growth inhibition and lowered the immunosuppression in a B16F10 melanoma model (52). In addition, the specific tumor targeting nanoparticles can possibly enhance the performance of antitumor immunity. For example, a pH and matrix metalloproteinase dual-sensitive micellar nanocarrier which can spatiotemporally control the release of anti-PD-1 and PTX in solid tumors has been developed by Su et al. (53). The report interestingly indicated that the PTX-induced immunogenic cell death (ICD) can activate the antitumor immunity along with the blocking of the PD-1/PD-L1 axis from anti-PD-1. Together, they hinder the immune escape of tumor cells due to PTX-induced PD-L1 up-regulation. Of note, the pH-sensitive polyethylene glycol (PEG) shell could be sheddable at the tumor acidity site resulting in release of anti-PD-1 and PTX. In general, polymer-based material can be utilized as a superlative and effective delivery system to sustain biocompatible antibodies and other immunological checkpoint inhibitors in cancer immunotherapy (**Figure 2**).

### 2.2 Chimeric Antigen Receptor-T Cells

#### 2.2.1 Monotherapy of CAR-T Cells

The term “adoptive cell transfer” (ACT) involves a group of cell-based anticancer immunotherapies and is very attractive for its smart and patient-tailored strategies (90, 91). ACT contains different steps to induce immune-mediated clearance of cancer. First, the circulating or tumor-infiltrating lymphocytes are collected from the patient. Then the cells are elected, activated *ex vivo*, genetically modified to express a cancer-targeting receptor, and multiplied to a therapeutic quantity. Finally, the cells are reinjected into the treated patient to recognize and eliminate cancer cells.

CAR-T cell is the most representative of ACT recently and has obtained optimistic clinical success. This therapy utilizes CAR to engineer autologous T cells for achieving immune activation without major histocompatibility complex (MHC)-restriction. The CAR is mainly composed of an extracellular antibody-derived antigen binding domain for cancer targeting and a one or more linked intracellular signaling domain. The extracellular binding domains are commonly constructed from single-chain variable fragments (scFvs) derived from tumor antigen-reactive antibodies (92). The intracellular signaling domain comprises CD3ζ chain domain and co-stimulatory domains, such as CD28 and/or 4-1BB to provide costimulatory signals for promoting CAR-T cell expansion, persistence, and function (93).

However, the manufacturing of CAR-T cell is time-consuming, expensive, and technically complex compared to other small therapeutic drugs. There are multiple aspects that need to be carefully controlled to archive a secure, therapeutically safe and effective CAR-T cell therapy, such as balanced CD4/CD8 ratio, the viability of differentiated CD3+CAR+ cells, and *in vitro* cytotoxicity and cytokine release against cells expressing the target antigen, which makes it costly and limits its widespread use (94). For instance, the list cost of Kymriah is US$475,000 and of Yescarta is US$373,000 which is higher than the current common cancer therapies’ cost (95). The manufacturing time to archive enough therapeutic cell numbers is from 3 to 4 weeks at least depending on different methods (96–98). It is noteworthy that the consistency of cell products plays a significant role in the tumor clearance effect of the patient. An optimum manufacturing of engineered T-cell process is required for reducing the cost and escalating clinical translation of CAR-T cell therapy.

Notably, utilizing engineered polymers can improve and streamline CAR-T cell manufacturing process. Diverse kinds of polymers have been synthesized and modified to apply in many medical engineering processes including cell culture (99), tissue engineering (100), separation (101), and drug and gene delivery (102). Similarly, many polymeric systems have been used to optimize streamlined CAR-T cell manufacturing process, mainly focusing on activation and genetic modification of the CAR-T cell.

#### 2.2.2 Polymeric Systems Utilized in CAR-T Cell Activation

The *ex vivo* CAR T-cell activation consists of three central signaling steps, which are T cell receptor (TCR) stimulation, CD28 co-stimulation, and cytokine signaling. Usually the T cell receptor (TCR) stimulation and CD28 co-stimulation utilize independent anti-CD3 and anti-CD28 monoclonal antibodies (mAbs) adhered to solid materials for receptor clustering. On the other hand, the cytokine signaling commonly uses soluble cytokines dissolved in the culture medium. Therefore, an activation platform should match the above requirements and these following criteria. The platform first needs to be “friendly” and can promote T cells to multiply to around 1 to 5 × 10^8^ CAR+ T cells for one patient (103), and maintain the stably therapeutic state *in vivo*. It is favorable that the platform can keep the balance number of CD4+ and CD8+ T cells in expansion (104). One more crucial point is the activation materials should be convenient to process and easily separate from T cells.

In this part, we discuss different polymeric T cells activation systems and their properties for adequately activating and expanding T cells.

##### 2.2.2.1 Nanoscale Activation Polymers and Particles

A commercial reagent to activate and expand human T cells *via* CD3 and CD28 was developed by Miltenyi Biotech which called it TransAct. According to the Miltenyi Biotech specification, TransAct has a nano core-shell structure where the core is iron oxide crystal and the shell is biodegradable polysaccharide matrix conjugated to humanized CD3 and CD28 agonists (105, 106). The size of TransAct is around 100 nm and can be filtered sterilized, the excess reagent can be cleared by centrifugation with the following conventional supernatant replacement or simply by a medium wash. Many studies have investigated and compared the effectiveness in expansion, differentiation, CAR transduction, and functions of activated T cells by TransAct and the results suggest that TransAct can be used for clinical-scale T-cell activation (107, 108).

In 2013, Mandal et al. synthesized a semiflexible synthetic dendritic cell for T cell activation (62). The semi-stiff poly(isocyano peptide) has been coupled with BCN-functionalized streptavidin (Sav) and then conjugated with biotinylated αCD3 antibodies to produce αCD3– “synthetic dendritic cells” (sDCs). The flexibility of this system allows the effector molecules on it which can effectively bind to the receptors of the targeted T cell. With the size around 150−200 nm, the αCD3–sDCs showed higher efficacy in expanding T cells compared to spherical αCD3–PLGA particles (1.8 μm diameter) or free αCD3 antibodies with the same antibody amount. The group then continuously developed this semiflexible synthetic mimic dendritic cells system with co-carrying anti-CD3 antibodies (αCD3) for triggering the T cell receptor and anti-CD28 antibodies (αCD28) as a costimulatory signal (109). The bifunctional αCD3/αCD28-sDC significantly stimulated T cell activation at a considerably lower antibody concentration than free soluble antibodies. Interestingly, the highest level of polyclonal T cell activation was only achieved when the sDCs carry both αCD3 and αCD28 antibodies on the same polymer. Utilizing different polymers αCD3-sDC and αCD28-sDC did not improve any T cell activation compared with αCD3-sDC alone. These results suggested that polymer flexibility and multiple signals equipped on one polymer are crucial for mimicking the endogenous receptor clustering needed for optimal T-cell activation.

##### 2.2.2.2 Microscale Artificial Antigen-Presenting Cells

On the other hand, different from nanoscale platforms, the microscale activation platforms imitate the original scale of endogenous antigen presenting cells and their immunological synapses with T cells. One of the first synthetic aAPCs design was based on latex (polystyrene) beads in the 1990s (110, 111). They have been used for exploring elemental features of T cell biology (112–114), and also as translational platforms for adoptive immunotherapy (115–118). These latex platforms are generated by chemically functionalized polystyrene surfaces with soluble proteins, or by binding avidin coated particles with biotin-labeled T cell activating proteins. Furthermore, MHC presented on the solid microparticle platforms by glutaraldehyde can induce stronger activation signal for T cells than the MHC presented on a cell membrane (119). Interestingly, hybrid lipid-latex particles, which utilize both the potential advantages of a solid particle and a flexible membrane, can be incorporated by coating polystyrene microsphere with plasma membrane vesicles (120–122). The studies suggested that this type of platform might effectively improve the efficacy of tumor immunotherapy with antigen. However, the main application of these polystyrene-based microparticle systems was for expansion of T cells *in vitro* because of its biodegradation and biocompatibility problems. The intravenous administration of larger 3-5 μm solid particles can accommodate in small capillary beds and cause capillary infarction (123, 124). Because of these issues, more advanced microparticle systems which are removable or biodegradable after the culturing period need to be developed.

In recent years, many biodegradable polymer-based microspheres have been utilized as vehicles for drug delivery (125, 126). The variety of these systems is varied in sizes in the range of hundreds of nanometers to 10 μm and can be generated from various polymers, such as poly(glycolic acid) (PGA), poly(lactic acid) (PLA), or their copolymer, poly(lactic-co-glycolic acid). After administration to the body, the biodegradable polymer particles are degraded to nontoxic substances and release the encapsulated drugs in several hours or weeks, depending on their design. Based on their favorable biocompatibility and biodegradability, the biodegradable polymer particles are an attractive candidate for aAPCs platforms that can deliver *in vivo* (127, 128). Notably, they can be formulated to release cytokine signals as they degrade and integrate these signals into designed aAPC (129, 130)

An ellipsoidal PLGA microparticle system was generated by Sunshine et al. as an aAPCs with 4.3 μm average diameter. Different aspect ratios have been investigated in T cells expansion efficacy (61). Compared with a spherical shape, ellipsoidal aAPCs dramatically improved T cell proliferation both *in vitro* and *in vivo*, especially at higher aspect ratios. The same tendency was observed with nanoscale aAPCs (131). On the aspect of surface topography, Fadel et al. developed an aAPC platform based on carbon nanotube−polymer composite (CNP) with the average size around 20−40 μm (132). The surface of the CNP possessed many defects that induced high surface area for attaching the stimuli for T cells. IL-2, a cytokine for T-cell cluster initiation and persistence after antigen priming, was encapsulated in biotinylated PLGA nanoparticles together with magnetite. These CNP aAPCs facilitated T cell expansion, differentiation, and the number of obtained T cells was proportionated to a level that would require at least 1000-fold less soluble IL-2 under conventional culture conditions. In addition, the magnetite in the PLGA nanoparticles allowed magnetic removal of CNP aAPCs, which contained the utmost physically and chemically stable carbon nanotubes.

#### 2.2.3 Polymeric Systems Utilized in CAR-T Cell Genetic Modification

Compared to viral transduction and nucleofection with expensive cost and safety issues, polymer-based nonviral gene delivery systems have recently become a potential candidate for CAR T-cell genetic modification. Cationic polymers which can electrostatically interact with negatively charged DNA and RNA to form polyplexes are the most favorable polymers for this purpose. Moreover, the positive charge of polyplex facilitates cellular uptake by the ionic interaction with negatively charged proteoglycans on the cell surface (133). Especially, if the cationic polymers contain amine groups, the amine groups will be protonated in the pH range of 5.0 to 6.8 and induce the proton sponge effect resulting in endosomal escape (134). Numerous polymers have been exploited such as poly(β-amino ester) (PBAE) (135), poly(2-dimethyl)aminoethyl methacrylate) (pDMAEMA) (136), polyamidoamine (PAMAM) (137), and branched polyethylenimine (bPEI) (138).

In this part, we report different polymer architectures for nonviral gene delivery to T cells, arranging them by *ex vivo* and *in situ* application. We also review main key barriers that require further improvement to achieve more efficient transfection with these systems.

##### 2.2.3.1 Ex Vivo Gene Delivery With Polymeric System

In 2018, Pun’s group investigated the transfection efficiencies of different synthesized polymers and their concomitant toxicity to T cells (139). They compared the transfection efficiency and cytotoxicity of branched polyethylenimine (bPEI), VIPER (virus-inspired polymer for endosomal release), linear pDMAEMA290, linear-branched (comb), and cyclic-branched (sunflower) polymers with varying pHEMA core sizes and pDMAEMA branch lengths in the Jurkat human T cell line. VIPER is an -block copolymer that consists of a hydrophilic cationic block for nucleic acid loading and colloidal stability, and a pH sensitive membrane lytic block for endosomal release in acidic pH (e.g., pH < 6.4) which was developed by their group (140, 141). Between the distinct polymer structures of pHEMA and pDMAEMA, sunflower and comb polymers obtained the most effective greatest transfection of Jurkat cells (25−50%) with low toxicity compared to bPEI, VIPER, or linear pDMAEMA290. The change in brand length of comb and sunflower polymer did not affect much in performance, however, reducing the core size of the comb polymer significantly lowers the transfection and cell viability. The best formula for the comb polymer was with core size of degree of polymerization 25 and branch length of degree of polymerization 16, this helped transfection efficiencies in primary T cells achieve an average 20% with mRNA and 10% with plasmid DNA (pDNA) and with cell viability persistently above 75%.

Many studies suggested that a decrease in branching of polymer can have a negative effect on transfection and viability. Christopher and Anja et al. developed multi-arm star-shaped pDMAEMA polycations for DNA transection and the result showed that the 5-arm star-shape exhibited lower cytotoxic than 3-arm counterparts with the similar transfection (142). Their research group continuously generated higher-branched star polycations, 20-arm pDMAEMA conjugated on a silsesquioxane initiator core and 120-arm self-assembled nanoparticles comprised of amphiphilic polybutadiene-block-pDMAEMA block copolymers showed 10 to 15% transfection efficiency of pDNA in primary T cells with more than 80% viability (143, 144). These data suggested that high polycation branching can be suitable for enhancing T-cell transfection. Notably, currently Yu et al. synthesized supramolecular self-assembled nanoparticles from adamantane-grafted PEG, adamantane-grafted PAMAM dendrimers, and cyclodextrin-grafted low-molecular-weight branched PEI which achieved more efficient pDNA transfection in Jurkat T cells than high-molecular-weight branched PEI (60).

Importantly, equipping cationic polymers with T-cell targeting ligands to facilitate cell surface binding and internalization could significantly enhance transfection. For example, Moffett et al. fabricated biodegradable poly(β-amino ester) (PBAE)-mRNA polyplex with poly(glutamic acid) (PGA)-modified antibodies (anti-CD3 or CD8) to form particular cell targeting mRNA nanocarriers (59). The nanocarriers successfully internalized into primary T cells within 2 h and effectively transfected more than 80% T cells with mRNA. Especially the lyophilized nanocarriers also showed the same result without any serve interference on T cell viability and expansion.

##### 2.2.3.2 In Situ Gene Delivery With Polymeric System

It is worth mentioning that for covering the expensive and laborious *ex vivo* CAR-T cell generation, *in situ* polymer-based gene delivery strategies have been developed. In 2017, Matthias T. Stephan’s research group utilized the polyglutamic acid (PGA)-conjugated antibody decorated poly(β-amino ester) (PBAE)-DNA polyplexes to target the T cell and genetically modify host T cells with leukemia-specific CAR genes *in vivo* (145). The nanocarrier has the core-shell structure, where the core was plasmid DNA complexed with PBAE polymers grafted peptides containing microtubule-associated sequences (MTAS) and nuclear localization signals (NLS) to facilitate nuclear plasmid import of their genetic cargo *via* the microtubule transport machinery under resting T-cell conditions. They used two plasmids, one with the leukemia-specific 194-1BBz CAR and one for coding the hyperactive iPB7 transposase for stable CAR integration. The shell of the nanocarrier was poly(glutamic acid) (PGA)-modified anti-CD3e f(ab′)2 for T cell targeting. The nanocarriers were systemically delivered into mice and the result indicated 34% (± 5.1%) of the circulating T lymphocytes bound CD3-targeted nanoparticles after 4 h, whereas the signals from off-target cells were 5.9 ± 2.8% after 4 h. To investigate the *in-situ* reprogramming circulating T cells ability of the nanocarrier system, five sequential doses of 3 × 10^11^ nanoparticles were intravenously injected into mice bearing B-cell acute lymphoblastic leukemia. Interestingly, only the injected nanoparticles co-encapsulated 194-1BBz and iPB7 transgenes groups showed rapidly and efficiently programmed peripheral T cells to recognize leukemia cells. After that, these T cells robustly replicated and differentiated to effector phenotypes while keeping a high-level expression of the CAR transgene over 24 d, then achieved a CD44^high^ CD62L^+^ memory phenotype. Remarkably, the tumor-inhibition profile of *in situ* nanocarrier-programmed CAR-T cells was commensurate with high-dose adoptively transferred CAR-T cells.

**Figure 3:**
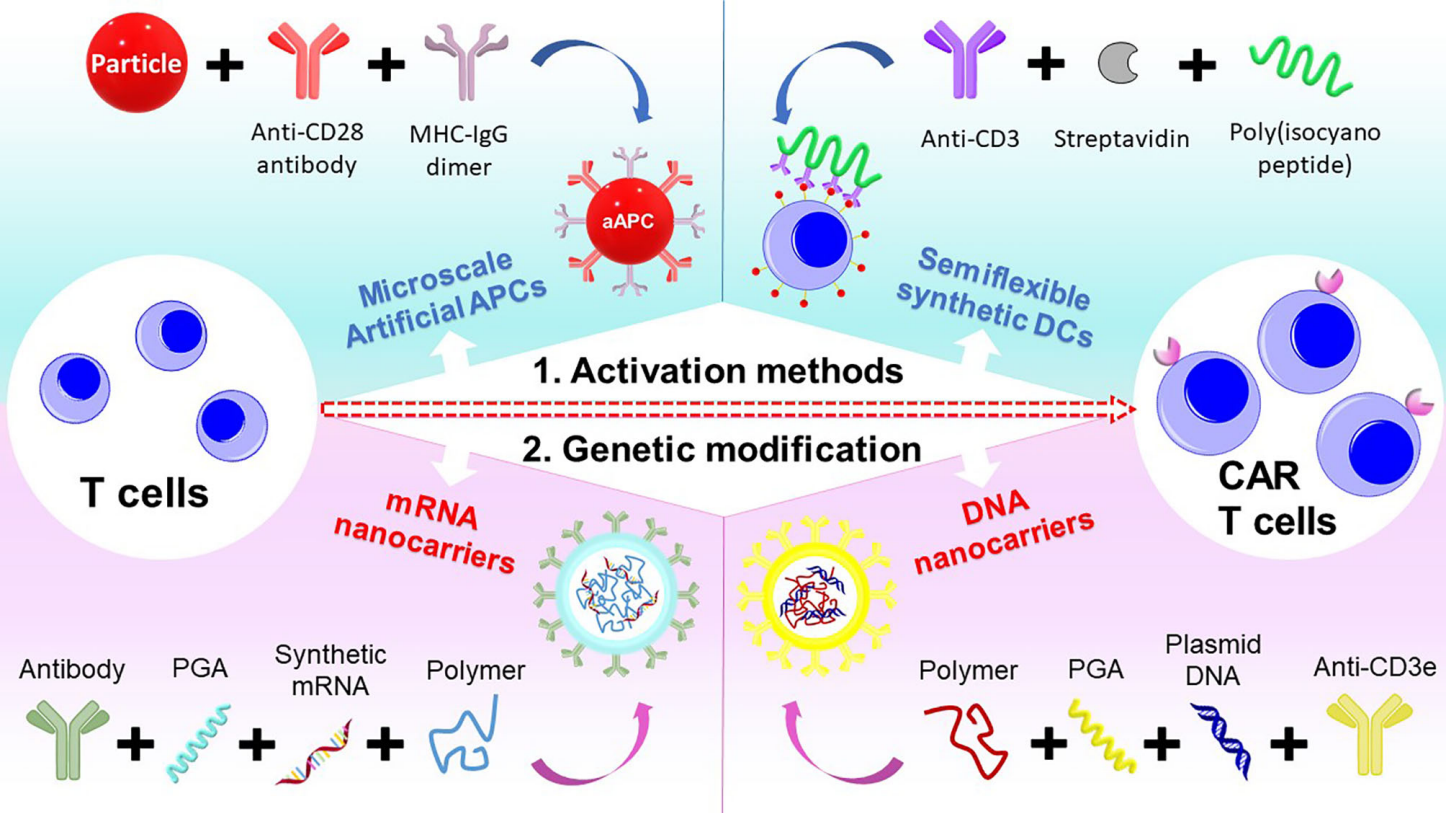
Schematic of different polymeric systems designed for CAR-T cell therapy.

In summary, the engineering and manufacturing of CAR-T remain a challenge for the widespread adoption of this technology. To overcome these challenges, we have introduced two main strategies using polymer systems to engineer T cells into CAR-T cells. As T cells need to be expanded for the engineering to CAR-T cells, novel biodegradable polymeric systems that act as synthetic dendritic cells can be utilized. Further, the use of branched cationic polymers and advanced polymeric nanocarriers can improve the genetic transfection of CAR genes into T cells (**Figure 3**).

### 2.3 Oncolytic Virus

#### 2.3.1 Monotherapy of Oncolytic Virus

Oncolytic virotherapy is the most auspicious access for tumor immunotherapy. The vital advantage of oncolytic virotherapy is based on the ability of replication-competent viruses that can proliferate selectively at tumor cells (146). Among many different viruses, adenoviruses can be represented as an example for illustrating oncolytic viruses as a whole. In 2005, the State Food and Drug Administration of China approved Oncorine, a replicative, oncolytic recombinant ad5 (rAd5-H101) for treating refractory nasopharyngeal cancer. This was considered the first approved oncolytic virotherapy for clinical use in the world (147, 148). Many types of cancer cells lose the p53 gene which causes drug resistance and lower survival rates in cancer patients (149). The p53 gene inactivation cell can halt the activation of apoptotic pathway. The Oncorine is a human serotype 5 adenovirus which is deleted by the E1B 55K gene. The elimination of the E1B 55K gene prohibits viral proliferation in normal cells, tolerating only multiples in the p53-lacking host cells. Therefore, the rAd5-H101 selectively proliferates in tumor cells and causes cancer cell lysis. The newly generated viruses release and infect surrounding cancer cells which leads to a chain-reaction of ultimate cancer cell destruction (150). The Oncolytic Adenovirus (oAd) recently become one of the most interesting generic immunotherapies for cancer in numerous phases of clinical trials (151, 152). The adenovirus (Ad) possesses several advantages that are beneficial for generic immunotherapy, such as the transduction ability of dividing and non-dividing cells with high efficacy, high loading capacity, easy modification of Ad genome, high production of viral progenies, and subsequent spreading of progenies to adjacent cancer cells (153–156).

It is noteworthy that the virus propagation inducing cell lysis is an extremely immunogenic process (157). This aspect is crucially relevant considering that the cancer cells usually disguise themselves from the host immune systems. The cell lysis releases multiple immunogenic molecules, such as abundant tumor-associated antigens for presenting to dendritic cells. The released virus genomes induce immunological danger signals through pathogen-associated molecular pattern (PAMP) and damage-associated molecular pattern (DAMP) receptors. These simultaneous actions stimulate the adaptive immune system, including helper CD4+ T cells and cytotoxic CD8+ T cells, toward the tumor resulting in disabling the tumor immunosuppression (158). Moreover, the T cell immunity toward the replicated adenovirus can enhance the overall antitumor process (159). The adenovirus infection can also indirectly activate the nature killing cells response for further immunogenic tumor inhibition (160). Indeed, many reports have investigated and confirmed the ability to inhibit the growth of different tumors of locally administered oAds, both in preclinical and clinical cases (161–166). The Oncorine is representative of oAds that have been used for clinical cancer treatment. However, the oAds still face certain challenges that narrow the therapeutic effectiveness for clinical trials.

One of the major hurdles for oAds’ efficacy is the pre-existing humoral immunity of the host body. Nowadays the human adenovirus serotype 5 (Ad5) is the most popularly used for adenoviral virotherapy and many reports showed that high percentages of the general population possess anti-Ad5 neutralizing antibodies (Nabs) which can easily terminate the bioactivity of Ad5. On the other hand, another utmost limitation of utilizing oAds for cancer therapy is the internalization of oAds dramatically depends on appropriate receptors, such as the Coxsackievirus and adenovirus receptor (CAR) on the surface of targeted cells. The CAR is a protein that belongs to a type I membrane receptor for subgroup C adenoviruses. CAR protein is expressed in many human tissues, including brain, heart, and some endothelial and epithelial cells (167–169). The efficiency of adenoviral transgene expression and CAR expression have been corresponded in numerous studies, suggesting that adenoviral binding and entry into target cells play an important role in achieving successful adenoviral gene expression (170, 171). Unfortunately, several cancer cell lines and clinical cancer tumors have been frequently observed in the loss of CAR expression, preventing attempts to achieve adequate oncolytic adenovirus virotherapy for cancer patients (172–175). It is impossible for oAds to obtain satisfactory remedial efficacy without overwhelming this CAR-dependent internalization. To overcome this, most currently ongoing clinical trials of oAds have been genetically modified to equip the beneficial fiber region. This adjusted fiber region can improve cellular internalization of the adenovirus and is independent of CAR expression level in heterogenic clinical tumor or tumor-specific internalization (176–178). However, processing genetic engineering for optimization of fiber-modified virus is risky and contains various disadvantages such as excessive cost, time, and labor-consuming. Inappropriate genetic editing can cause viral replicability loss, viral inactivity, and inadequate gene sequence expression (163).

**Figure 4:**
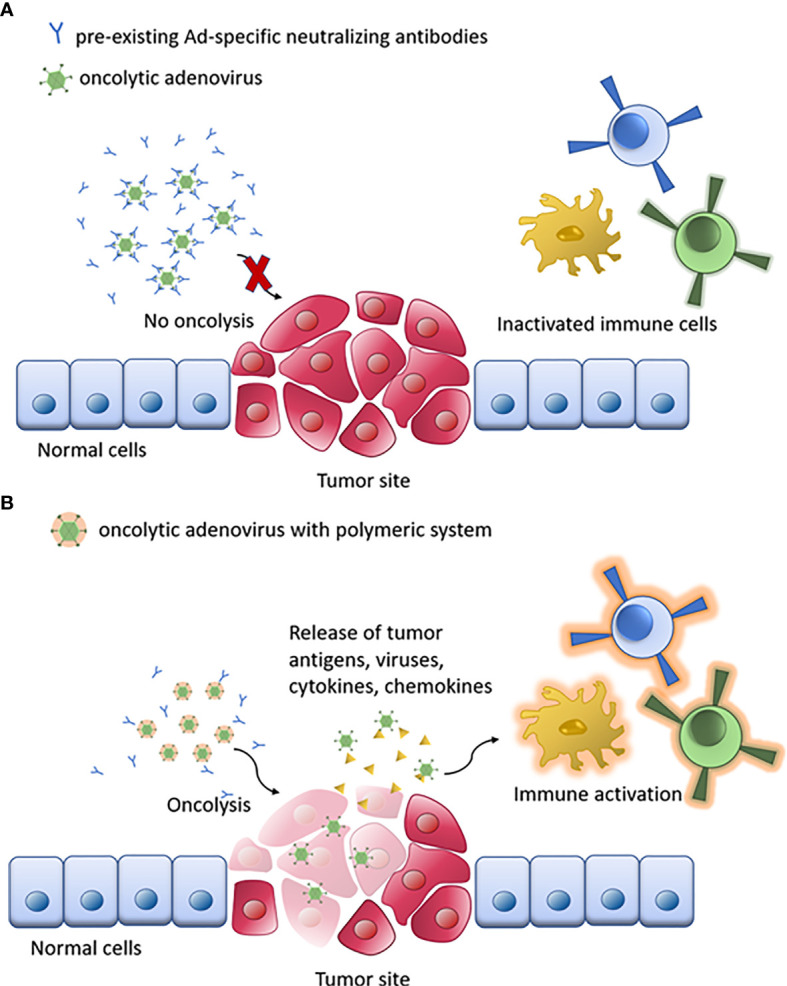
Schematic of oAd immunotherapy with polymeric system. **(A)** The naked oAd is disabled by pre-existing Ad-specific neutralizing antibodies. **(B)** Oncolysis by polymer/oAd system stimulates the immune system response against tumor cells, enhancing the therapeutic response.

To overcome these severe limitations of both local and systemic administration of oAd, many advanced polymer-based delivery techniques have been developed in recent years. The polymer-based delivery techniques can enhance the bioavailability while at the same time provide the necessary (**Figure 4**) protection of oAd. The delivery and tumoral targeting profile of encapsulated oAd can be adapted to the specific medical goals by choosing a proper encapsulation polymer (179, 180). By covering the outer surface of oAd, the polymer-based delivery systems can avoid adverse problems caused by the viral capsid. Furthermore, the applied materials can sufficiently equip the oAd with beneficial properties and override the natively disadvantageous attributes of viral vectors, which efficiently contribute to improving the tumor-specific accumulation of oAd (58, 181–183) (**Table 2**).

**Table 2:** Summary of recent research on different polymeric systems for oAd.

Methods	Polymer systems	System properties^a)^	Oncolytic adenoviruses	Cancer cellmodel	Efficacy	Ref.
Physical interaction	Multidegradable bioreducible core-cross-linked polyethylenimine (rPEI)	D: 192.8nmZ: 24.3mV	RdB/shMet	MCF7, A549, HT1080	Improved transduction efficacy and achieved CAR-independent cell internalization.	(54)
Physical interaction	Bile acid-conjugated poly(ethyleneimine) (DA3)	D: 324 ± 3.08 nmZ:10.13 ± 0.21mV	RdB-KOX	HT1080	Hindered tumor angiogenesis and enhanced anti-tumor efficacy	(184)
Physical interaction	Methoxy poly(ethylene glycol)-b-poly{N-[N-(2-aminoethyl)-2-aminoethyl]-L-glutamate} (PNLG)	D: 130-140 nmZ:~19 mV	Ad-DB7-U6shIL8	HT1080, A549	Highly enhanced tumor accumulation and anti-tumor efficacy, preserved bioactivity of Ad at 37°C	(185)
Physical interaction	mPEG-PEI-g-Arg-S-S-Arg-g-PEI-mPEG(PPSA)	D:~200 nmZ:19.6 ± 0.9 mV	DWP418	MCF7	Increased transduction efficacy and obtained CAR-independent cell internalization, improved anti-tumor efficacy	(55)
Chemical interaction	Polyethylene glycol (PEG)	D:122.8-138.5nmZ:19.6 ± 0.9 mV	Ad-GL	Hep3B, LNCaP	20-kDa PEGylation of oAd reduced transduction of the liver and toxicity, improved anti-tumor efficacy	(56)
Physical interaction	Poly(amidoamine) dendrimer (PAMAM)	No data	Ad5-CMV/NIS	HCC	Lowered hepatic accumulation, significantly delayed tumor growth and extended survival	(186)
Physical interaction	Amphiphilic polyphenylene dendrimer (PPD)	D:~200nmZ:~-40 mV	Ad5	CHO-K1	Increased internalization into CAR-negative cells and introduced new concepts and a possibility for binding cancer cell targeting groups	(57)
Physical interaction	poly(CBA-DAH)-PEG-RGD	D: 267.6 ± 54.8 nm	ΔDB7-U6shIL8	HT1080, MCF7	Increased both transduction and achieved CAR-independent, only need integrins for targeting cancer cell transduction	(182, 187)
Physical interaction	Chitosan–PEG–folic acid	D:~140 nmZ: 2.1 mV	Hmt	KB	Targeted and increased tumor accumulation at folic acid receptor overexpress cancer cell model, increase the anti-tumor efficacy	(58, 181)

^a^)D, diameter; Z, Zetapotential.

#### 2.3.2 Combination Oncolytic Adenovirus Therapy With Polymer Systems

##### 2.3.2.1 Overcoming CAR-Dependent oAd Uptake by Cationic Polymers

The Ad surface interestingly possesses negative charge (188, 189). Therefore, the anionic surface of Ad can electrostatically interact with the cationic polymer to form an Ad/polymer complex. Because of the cationic charge of the polymer, the complex surface is positive, therefore, the complex can enhance the cellular uptake and transgene expression of Ad. Various kinds of polymers have been used for complexing with Ad, most of them based on the positively charged amine groups in the backbone of the polymers. It is noteworthy that the secondary and tertiary amines additionally have high buffering capacity, which compellingly promotes the escape from endosomes into the cytosol of the virus due to the proton sponge effect (190, 191). The polymer structure can be beneficially designed and easily controlled to obtain the advanced bio-function for improving oAd tumor-inhibition efficacy. The method to generate oAd/polymer complex is also straightforward and effortless compared to genetic editing or chemical modification of the virus structure. The oAd/polymer complex can be formed in an aqueous buffer without any additional steps or chemicals. Notably, the original bioactivity of the oAd does not change and is preserved in the complex. On the other hand, the remaining limitations of this method are that the cationic oAd/polymer complex not only can specifically internalize to the cancer cells but also uptake to the body’s healthy cells, subsequently increasing cytotoxicity toward the body. Moreover, the electrostatic interaction of the complex can easily be dissociated in the bloodstream through intravenous injection *via* associating the cationic polymer with the negatively charged serum protein. The association with some specific serum protein can also trigger the interaction with macrophages and monocytes (192, 193).

###### 2.3.2.1.1 Engineered Poly(Ethyleneimine) for oAd

In the history of developing transfection reagents, beside the polylysine, the poly(ethyleneimine) (PEI) was the second polymeric transfection agent discovered (194–196). The repeating unit of PEI consists of the amine group and two carbon aliphatic spacers. Depending on whether the PEI is linear, branched, or dendrimer form, the structure of PEI can contain primary, secondary, or tertiary amino groups. The PEI (25 kDa) is considered as a standard model for transfection reagent because of its high transgene expression (197, 198). Though the PEI performs high cytotoxicity by two mechanisms, the cationic charge of PEI can possibly disrupt the cell membrane and lead to necrotic cell death or disrupt the mitochondrial membrane after cytosol internalization leading to apoptosis. Many attempts have been made to reduce the PEI cytotoxicity, including modified with non-toxic polymers such as polyethylene glycol (PEG) or other biopolymers, or cholesterol (199–203). Among these attempts, various bioreducible PEIs have been studied for applying cancer therapy (204–206). The bioreducible PEI contains the disulfide moiety in the copolymer blocks, which will degrade *via* a reductive environment in the cytoplasm and release the therapeutic materials. The degraded fragments can be clearly excreted by the host body’s excretory system producing low systemic cytotoxicity. In 2015, a new low molecular weight PEI multi cross-linked to bioreducible disulfide cystamine core (rPEI) had been generated by Choi et al. and complexed with Ad (54). The Ad/rPEI complex showed remarkably higher transduction efficiency compared to naked Ad in both CAR-positive and -negative cancer cells, which suggests that the complex can independently transduce to CAR expression cancer cells. Moreover, the GFP intensity of GFP-expressing Ad in the 16kDa rPEI complex was manifold higher than Ad/25 kDa PEI in all A549 (7.7-fold), HT1080 (2.9-fold), and MCF7 cells (2.0-fold), which exhibited the remarkable transduction efficiency of the Ad/rPEI. The oncolytic Ad expressing short hairpin RNA against c-Met mRNA complexed with rPEI, demonstrated more efficient cancer cell killing effect, suppression of Met and VEGF level, and viral production than naked Ad. In recent times, Lee et al. developed a bile acid-conjugated 1.8 kDa PEI (DA3). The VEGF inhibitory gene (KOX) expressing-oncolytic Ad was complexed with DA3 (KOX/DA3) and showed a higher transduction efficiency than naked oAd in both CAR-positive and -negative cancer cells (184). Interestingly, the internalization mechanism of the Ad/DA3 complex and naked Ad were investigated and the results indicated that the mechanism of cellular uptake of the Ad/DA3 complex differed from that of naked Ad. The Ad/DA3 complex appeared to be transduced *via* clathrin-, caveolae-, and macropinocytosis-mediated endocytosis, whereas the naked Ad appeared to internalize cells mainly by clathrin-mediated endocytosis. The KOX/DA3 exhibited an improved antitumor efficacy compared with naked KOX. The data suggest the DA3 can facilitate the amplification and active replication of KOX.

###### 2.3.2.1.2 Biodegradable/Reducible Polymers Coated oAd

In 2013, a biodegradable methoxy poly(ethylene glycol)-b-poly{N-[N-(2-aminoethyl)-2-aminoethyl]- L-glutamate (PNLG) polymer was generated and used to coat oncolytic Ad (Ad-ΔB7-U6shIL8) (oAd/PNLG) by our group (185). The GFP-expressing Ad complexed PNLG showed improvement in transgene expression in both positive and negative CAR-expressing cells than naked Ad and Ad/bPEI *in vitro*. In addition, the oAd/PNLG showed better cancer cell killing efficacy *in vitro* than naked Ad, Ad/PEI. The biodistribution result demonstrated higher tumor accumulation when systemically administered oAd/PNLG compared with naked Ad and Ad/PEI. The oAd/PNLG showed 1229-fold higher tumor-to-liver ratio than the naked oAd. It is noteworthy that the oAd/PNLG also showed significantly lower innate and adaptive immune responses than the naked Ad. Another cationic polymer was introduced by our group in 2014 especially containing arginine moieties that enable promotion of cellular internalization in both low and high CAR-expressing cells (55). The polymer, mPEG-PEI-g-Arg-S-S-Arg-gPEI-mPEG (PPSA), contains multiple arginine functional moieties for increasing transgene expression and introduced a bioreducible disulfide bond to lower cytotoxicity. The oncolytic Ad (DWP418) was complexed with PPSA (DWP418/PPSA) and intratumorally injected into CAR negative MCF7 xenograft mice. The result revealed that the DWP418/PPSA provided more effective anti-tumor responses compared with naked DWP418. Moreover, the results also indicated the DWP418/PPSA-treated mice produced less innate immune response and oAd-specialized neutralizing antibodies than the only DWP418-treated group but produced more viral replication and viral cancer cell lysis in tumor tissues. The optimistic results demonstrate the advantages of utilizing the bioreducible and biodegradable polymer masked oAd in cancer treatment.

##### 2.3.2.2 Tumor Targeting by Oncolytic Ad/Polymer

On the other hand, the complexes of oAd with cationic polymer still face diverse obstacles for clinical translation, such as the cationic oAd/polymer complex not only non-specifically internalizing the cancer cells but also uptaking the normal tissues, subsequently causing cytotoxicity. The electrostatic interaction of the complex can also easily be dissociated in the bloodstream through intravenous injection *via* associating the cationic polymer with the negatively charged serum protein (207–209).

###### 2.3.2.2.1 PEGylation of Oncolytic Ad

Polyethylene glycol (PEG) is a biocompatible, synthetic, hydrophilic polyether, composed of CH_2_CH_2_O repeat units. Abundant therapeutic drugs are frustrated by short half-lives *in vivo*. The PEG is considered the first material that successfully improves both physiochemical, pharmacodynamic, and pharmacokinetic properties of biological drugs such as proteins, peptides, enzymes, synthetic drugs, etc. and has been approved by the FDA in 1990 (210–212). By interacting with PEG, the therapeutic agents can increase water solubility, and reduce renal clearance and immunogenicity. In 1999, the first research on conjugating Ad with PEG was recorded (212). The PEGylation did not show any damaging effect on the bioactivity of Ad. Also it was shown that it can enhance serum stability, blood circulation, and tumor-specific accumulation. The PEGylation has been proved to improve remarkably the Ad to avoid antibody neutralization and also unfavorable immune response *in vitro* and *in vivo* (213, 214).

In 2009, Doronin et al. investigated the effect of PEGylation on oncolytic Ad by using 5-kDa and 20-kDa PEG (56). Interestingly, the therapeutic efficacy of PEGylated oncolytic Ad improves with increasing molecular weight of the PEG. The 20kDa PEG conjugated oAd dramatically reduced liver accumulation and hepatotoxicity by systemic administration. Compared with naked and 5-kDa PEGylated oAd, the 20-kDa PEGylated oAd showed reduction hepatocyte transduction by 19- or 90-fold, respectively, in hepatocarcinoma xenograft tumor models. Moreover, the 20-kDa PEGylated oAd administered mice possessed average survival rate double to only the naked oAd group. The effectiveness of higher molecular weight PEGylated oAd can be explained by PEGylated oAd that has a higher molecular weight and larger hydrodynamic radius than the naked oAd and cleared from the body at a much slower rate by kidney or Kupffer cells in the liver, subsequently increasing the half-life and passive tumor targeting through EPR effect. These positive advantages promise that high molecular weight PEGylation can benefit the therapeutic and survival efficacy of oncolytic adenovirus.

###### 2.3.2.2.2 Dendrimer-Coated Ad

Dendrimers are highly ordered, symmetric, branched polymeric molecules. Many reports have shown that dendrimers potentially transfer genes into cells without damaging or deactivating the DNA (215–217). Poly(amidoamine), or PAMAM, is the most popular dendrimer, which used ethylene diamine or ammonium as a core molecule. In 2013, amine-terminated generation 5 PAMAM dendrimer was used to coat sodium iodide symporter (NIS) expressing oncolytic Ad (Ad5-CMV/NIS) (186). This research conducted by Gruanwald et al. showed the complex increased the transduction efficacy in CAR-negative cells and preserved the activity of Ad against neutralizing Abs. Moreover, the ^123^I scintigraphy of mice from biodistribution results demonstrated systemically administered PAMAM-complexed Ad5-CMV/NIS significantly diminished transgene expression and induced lower liver toxicity than naked Ad5-CMV/NIS. Further, the *in vivo* antitumor study indicated the PAMAM-complexed Ad5-CMV/NIS possessed higher tumor inhibition efficacy and survival rate than the naked Ad. Through the research, the PAMAM complex displayed improvement in tumor targeting and reducing liver accumulation of oAd, therefore suggesting potential application of oncolytic virotherapy by systemic administration. Interestingly, in 2020 Wagner et al. reported an amphiphilic polyphenylene dendrimer (dendron) to complex with adenovirus which contains a propargyl-modified triethylene glycol linker at the core (57). This linker of the dendron provides for the complex system high aqueous solubility and the possibility to introduce chemical modifications on the viral surface without directly covalently modifying the virus particles. The research showed that the dendrons link to the surface of the adenovirus through their polar and nonpolar surface groups. The report indicated the masking dendrons can promote the internalization of the Ad/dendron complexes into CAR-negative CHO-K1 cells. Even though the research did not proceed any further *in vivo* experiments, it did introduce a new concept and a possibility for binding cancer cell targeting groups, subsequently expanding the therapeutic potential of oAd.

###### 2.3.2.2.3 Oncolytic Ad Complexed CD-PEG-cRGD

Even though utilizing cationic polymer masking oAd can improve the therapeutic efficacy, there are still several limitations for the clinical setting. Some nano complexes have low diffusion and the positive charge of the complex can induce normal cell internalization or interact with RES, leading to low tumoral accumulation (207).

Our group has developed an Arg-Glye-Asp (RGD) peptide domain conjugated poly(cystaminebisacrylamine-diaminohexane) [poly(CBA-DAH)] (CD) for modifying oAd to target the tumor side (187). The new biodegradable polymer CD possesses the disulfide bonds which allow the polymer to be smoothly cleaved into harmless fragments when reaching the reductive environment of the cytoplasm. The cyclic RGD, on the other hand, can specifically target avb3 and avb5 integrins which were overexpressed in abundant types of tumor cells (218, 219). The oAd/cRGD-conjugated CD complex increased both transduction and cancer cell killing effect with high specificity in a dose-dependent manner *in vitro*. The competition assay, which used anti-CAR and anti-integrin antibodies, demonstrated that the oAd/cRGD-conjugated CD complex achieved CAR-independence and only needed integrins for targeting cancer cell transduction, contracted to naked Ad which needs both of CAR and integrins to infect. Furthermore, the oAd/cRGD-conjugated CD complex also showed dramatically induced apoptosis and necrosis besides reduced VEGF and IL-8 secreting in cancer cells compared to naked oAd. The *in vivo* antitumor efficacy of the oncolytic Ad/CD-PEG-cRGD complex was further reported in a lung orthotopic tumor model in 2014 (182). The oAd was successfully covered by the bioreducible CD-PEG-cRGD polymer help to avoid anti-viral immune responses resulting in decreased hepatotoxicity. More importantly, the length of PEG moiety showed a large impact on the therapeutic effect of the system. The CD-PEG_2000_-cRGD, which has 2000 Da PEG length, coated with oAd obtained a better tumor growth inhibition than the CD-PEG_500_-cRGD coated oncolytic Ad, illustrating that PEG with longer length could improve the pharmacokinetic and tumoral accumulation of the complex. These reports have indicated the cRGD and CD-PEG-cRGD potentially enhanced the transduction and tumoral accumulation of the oAd according to the interaction between tumor homing peptides and integrins.

###### 2.3.2.2.4 Folate Receptor Overexpressed Cancer Therapy With Oncolytic Ad/Chitosan PEG-FA

In recent years, folic acid (FA) has been wildly investigated as an active targeting moiety for cancer therapy according to a large number of cancer cells overexpress folate receptors on their surface (220–222). To utilize the tumoral targeting property of FA, our group has developed an oAd/chitosan-PEG-FA complex by the advanced electrospinning technique (58, 181). After the electrospinning process, the chitosan-PEG-FA coating on the Ad was confirmed by the size and the ζ-potential of the complex as well as the biological activity of the Ad was preserved. Furthermore, the oAd/chitosan–PEG–FA obtained the blood retention time 48.9-fold higher than the naked oAd and the liver uptake was also 378-fold reduced. This suggested the PEG moiety can lower the non-specific liver uptake dramatically. It is noteworthy that the oAd/chitosan–PEG–FA significantly increased the tumor-to-liver ratio by 1.08 × 10^5^-fold compared to naked oAd. This strongly exhibited the excellence in tumor selectivity of oAd/chitosan–PEG–FA complex *in vivo*. The antitumor efficacy result confirmed the oncolytic Ad/chitosan–PEG possessed more novel therapeutic effect than the oncolytic Ad/chitosan–PEG.

##### 2.3.2.3 Combined With Immune Cell Therapy by Polymeric Hydrogel

Another interesting utilization of a polymeric system is hydrogel. The hydrogel is the polymer matrix which contains a high amount of water or biological fluid. Therefore, the hydrogel can be utilized to deliver several types of therapeutic agents, including cells or viruses. One researcher of our group in 2017 applied gelatin-hydroxyphenyl propionic acid (GHPA)-based hydrogel to co-deliver oAd and dendritic cells (DCs) for combined immunotherapy (223). The DCs can present TAA to cytotoxic T cells to induce tumor-specific immunity while the oAd can co-express interleukin (IL)-12 and granulocyte-macrophage colony-stimulating factor (GM-CSF) to elicit synergistic tumor growth inhibition. The hydrogel system successfully protected the biological activity and released both oAd and DCs in a controlled manner, leading to a long retention time of both therapeutics in the tumor site. Moreover, the expression level of IL-12, GM-CSF, and interferon-γ (IFN-γ) in the tumor treated with oAd- and DC-loaded gel (oAd + DC/gel) was dramatically higher than oAd or DC only, or the dual injection without gel (oAd + DC). As a result, the number of both activated endogenous and exogenous DCs, the number of DCs migrated to lymph nodes, and the tumor infiltration of CD4+ and CD8+ T cells are remarkably high in the (oAd + DC/gel) samples. Further, the tumor inhibition profile of the (oAd + DC/gel) group indicated the best antitumor performant which demonstrated the novelty of this method. By utilizing gelatin-based hydrogel, the research showed the potential of co-delivery oAd and DCs to the tumor tissue not only can preserve but also induce synergistic immune response with a single dose for a relatively long time.

In summary, systemic delivery of oAd has shown limited therapeutic efficacy due to hepatotoxicity, immunogenicity, and CAR-dependent transduction of Ad. Therefore, surface modification of oAd with polymeric systems provides a novel delivery strategy which can reduce immunogenicity, nonspecific liver sequestration and hepatotoxicity, and enhance transduction efficacy. Furthermore, it prolongs blood retention time and enhances overall intratumoral accumulation of Ad, ultimately leading to potent therapeutic efficacy.

## 3 Conclusion and Perspectives

Cancer immunotherapies using ICI, CAR-T, and OV are very new and promising treatment strategies to eradicate tumors and inhibit tumor metastasis by activating the immune system. These strategies can be considered as a game-changer for modern cancer treatment in the next coming time. Despite abundant advancements and excellent clinical outcomes, many challenges are remaining and need to be overcome, relating to low antitumor efficiency, costly processes, and side effects (224, 225). In this review, we have summarized divergent polymeric systems for improving the overall therapeutic efficacy of mentioned cancer immunotherapies. Through utilization of appropriate polymeric systems, the above-mentioned disadvantages of immunotherapies can be resolved and can further enhance therapeutic efficacy, biocompatibility, and high specificity. Polymeric systems provide a novel delivery method with numerous benefits such as low toxicity, excellent biodegradability, and flexible surface and size modification for the conjugation of immune ligands and the loading of immunotherapeutic agents. It is notable that polymeric systems can protect and preserve the bioactivity of bioactive agents, insulating them from the unfavorable immune reaction or stimulate the favorable one in the body condition.

Regardless of these polymeric systems-mediated delivery of immunotherapy methods, there are still hurdles that remain before the application to patients in the clinic, such as low treatment efficacy, resistance to cancer immunotherapies, patient safety issues, and expensive treatment costs. Therefore, further research must be conducted to improve current delivery strategies. Delivery systems must yield a more effective and reliable approach for the delivery of immunotherapy agents. New methods to proliferate and engineer immune cell therapies *ex vivo* should also be developed with lower-cost manufacturing methods. For these purposes, it is expected that in the future, polymeric systems will be more extensively and ingeniously fabricated for cancer immunotherapies, hence enhancing their efficacy, and lowering immune-related side effects. Moreover, for the development of the clinical translation of nanomedicine, extra investigation on whole-body biocompatibility and the effects of various polymer systems on different organs is necessary. Furthermore, the next generation of polymer nanotechnology-based immunotherapy should supremely possess multiple functions, including targeting capability, smart responsiveness, and convenient applicability. Especially the personalized immune treatment with the assistant of polymer nanoparticles will be a critical and promising research trend in the future.

## Author Contributions

Conceptualization, TL, A-RY, and C-OY. Methodology, TL. Formal analysis, TL, A-RY and TT. data curation, TL and TT. Writing—original draft preparation, TL and A-RY. Writing—review and editing, TT and C-OY. Supervision, C-OY. Funding acquisition, C-OY. All authors have read and agreed to the published version of the manuscript.

## Conflict of Interest

C-OY is CEO of GeneMedicine, Co., Ltd.

The remaining authors declare that the research was conducted in the absence of any commercial or financial relationships that could be construed as a potential conflict of interest.

## Publisher’s Note

All claims expressed in this article are solely those of the authors and do not necessarily represent those of their affiliated organizations, or those of the publisher, the editors and the reviewers. Any product that may be evaluated in this article, or claim that may be made by its manufacturer, is not guaranteed or endorsed by the publisher.
